# Lab life, seasons and chromosome fusions affect non-cell-autonomously proliferation and neurogenesis, but not oligodendrogenesis, in mice and voles

**DOI:** 10.1038/s41598-025-01670-3

**Published:** 2025-05-28

**Authors:** Athanasia Rapti, Theodosia Androutsopoulou, Evangelia Andreopoulou, Maria Mellou, Georgios Leventakos, Maria Anesti, Konstantina Mastori, Myrto Chatzopoulou, Paraskevi Smyrli, Nikiforos Lakos, Kawthar Muse, Georgios P. Mitsainas, Ilias Kazanis

**Affiliations:** 1 Lab of Developmental Biology, Department of Biology, University of Patras, 26504 Patras, Greece; 2 Section of Animal Biology, Department of Biology, University of Patras, 26504 Patras, Greece; 3 School of Life Sciences, University of Westminster, London, UK

**Keywords:** Glial stem cells, Neural stem cells, Adult neurogenesis

## Abstract

Environmental and behavioral factors have been shown, in experimental settings, to affect neurogenesis in the mouse brain. We found that the density of proliferating neural stem/progenitor cells (NSPCs) and of neuroblasts was significantly lower in the Subependymal Zone stem cell niche of lab mice when compared with mice and pine voles captured in the wild, with seasonal variation observed only in voles. Moreover, levels of proliferation and neurogenesis were found to decrease in proportion to the decrease in the numbers of chromosomes (from the typical 2n = 40 down to 2n = 26) caused by Robertsonian fusions. In contrast, oligodendroglial progenitors and microglial cells were unaffected by wildlife, seasons and chromosomal fusions. When NSPCs were grown in cultures no differences were detected, suggesting that environmental and genetic effects are mediated by non-cell-autonomous mechanisms. These “real-world” data provide a platform for the identification of systemic factors and genetic loci that control postnatal brain neurogenesis.

## Introduction

**Fig. 1:**
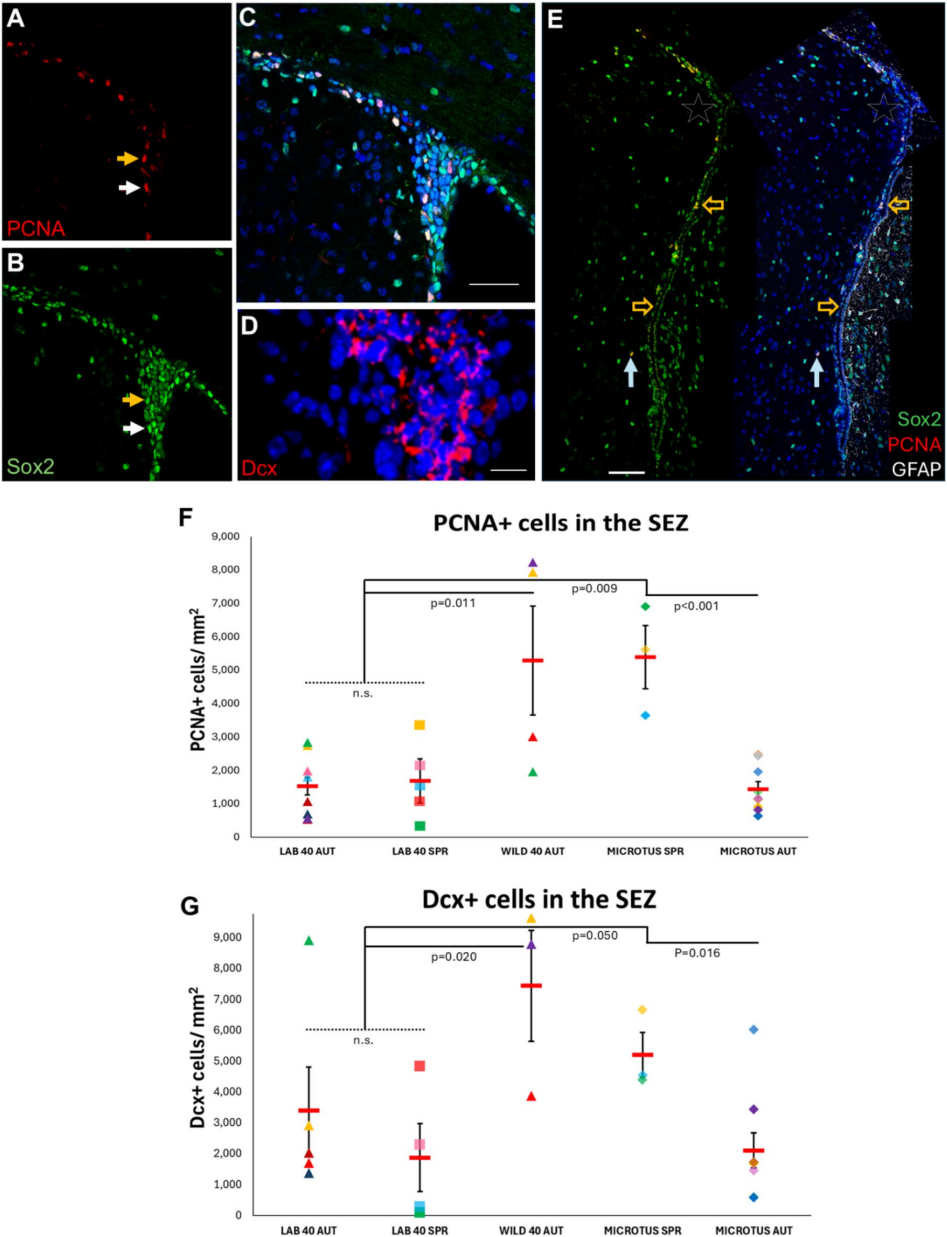
Effects of habitat and species on proliferation and neurogenesis in the SEZ niche. (**A**–**C**) Microphotograph of the “dorsal horn” of the lab mouse SEZ, after immuno-fluorescence on brain sections for PCNA (nuclear staining, in red, in **A**) and Sox2 (nuclear staining, in green, in **B**). Merged image in **C**. [Scale bar: 100 μm. The yellow arrow indicates a PCNA + cell with low Sox2 immunoreactivity. The white arrow indicates a PCNA + cell with high Sox2 immunoreactivity] (**D**) Detail of the “dorsal horn” of the SEZ in a Wild^2n=40^ mouse showing Dcx + cells (cytoplasmic staining in red). [scale bar: 15 μm]. (**E**) Image of the total dorsoventral SEZ area in a *Microtus thomasi* individual (autumn collection) after immunostaining for Sox2 (in green), PCNA (in red) and GFAP (in white). The SEZ has a typical structure for rodents (mice and rats), with Sox2 expression in ependymal cells (indicative examples of ependymal cell groups indicated with yellow arrows). An “ectopic” PCNA + cell, located deeper in the striatum, is indicated with a white arrow. Note that the density of PCNA + cells is significantly decreased at the “dorsal horn” area, indicated by the white star (compare with the area shown in **A**). [scale bar: 50 μm]. (**F**–**G**) Scatter plot graphs of the density of PCNA + and of Dcx + cells according to life conditions (lab or wild), species and season. [Individual values are shown with different colours and shapes, as explained in Suppl. Fig. 1. Mean values are shown with red lines and error bars depict the SEM. Comparisons between groups were performed using one-way ANOVA, followed by Tukey’s *post-hoc* analysis. “n.s.”: not significant].

In mammals, postnatal brain neural stem and progenitor cells (pbNSPCs) cluster within specialized microenvironments called stem cell niches. They remain in quiescence and infrequently transit towards mitotic activation, giving rise to transit amplifying progenitors that subsequently generate committed neuronal or glial progenitors^1–3^. A well-described niche in rodents and humans is located at the subependymal zone (SEZ) of the lateral ventricles (also known as ventricular-subventricular zone)^4^. In mice and rats, the bulk of the SEZ niche is located in a narrow layer of cells adjacent to the ventricular ependyma, at the striatal side of the lateral ventricles, and can be identified based on the expression of markers of cell proliferation, such as Proliferating Cell Nuclear Antigen (PCNA) (Fig. 1A,C) and of immature neuronal identity, such as Doublecortin (Dcx) (Fig. 1B)^5,6^. A distinct pool of brain progenitors, called Oligodendrocyte Progenitor Cells (OPCs), can be also detected based on the co-expression of proliferation markers and markers of oligodendroglial lineage identity, such as Olig2 or PDGFRα^5,7,8^. OPCs are scattered throughout the brain parenchyma, with the corpus callosum (CC) being a white matter tract particularly rich in them^9–11^.

A wide range of ecological and behavioral factors have been shown, in lab settings and each one separately, to control the behavior of pbNSPCs, including: pregnancy^12^, physical exercise^13,14^, stress^15^, environmental enrichment^16,17^, social interaction^18^, night/day duration^19^, olfactory stimuli (for the rodent SEZ)^20^ and the microbiome^21^. An even wider list of diffusible (local and long-range) molecules, cell-to-cell signals and metabolic factors have been, again experimentally, proposed to regulate pbNSPCs^4^. Such experimental animal work is important in basic research, but can be less valid for translational biomedical research, as it relies on the use of animals maintained and handled in highly controlled conditions and with the role of each factor explored individually. Hence, it is imperative to dissect mechanisms and factors that regulate pbNSPCs in wild rodent populations, the life of which integrates the multiple parameters listed above. To achieve this, it is necessary to identify appropriate wild animal comparator populations that -in the absence of experimental manipulation and easy access to large animal numbers- will enable the link of descriptive information with genetic and metabolic data.

As a first step towards this direction, we generated “real world” data comparing neurogenesis and oligodendrogenesis in the SEZ and the CC in brain samples obtained from laboratory and wild populations of *Mus musculus domesticus* (house mouse). To increase the strength of the analysis, we enriched our samples in two ways: firstly, by including a fossorial species, the *Microtus thomasi* (Thomas’s pine voles), which is found in open fields and was captured in underground burrows (Supplemental Fig. 1 and Supplemental Table 1). Secondly, we sampled populations of wild mice with karyotypes that deviate from the typical 2n = 40 due to the naturally occurring phenomenon of Robertsonian fusions (Rb). As the typical mouse chromosomes are acrocentric, the fusion of two of them can result in the formation of metacentric chromosomes^29^, leading to established mouse populations with lower numbers of chromosomes, down to 2n = 26^22^. Rb lead to the reorganization of chromatin; albeit, without major loss of genetic material^23^. So far, there is no evidence of phenotypic alterations caused by Rb in mice^24^; however, this phenomenon leads to higher levels of genetic differentiation through effects to meiotic recombination^25^ and is regarded as a speciation factor because it leads to the genetic isolation of mouse populations by inhibiting the flow of genetic material^26–28^ and has been linked to infertility in humans^29^.

We focused our analysis on the SEZ, which contributes to olfaction and is positioned and structured in a way that allows the integration of multiple, peripheral and local, stimuli derived via the cerebrospinal fluid, the vasculature, astrocytic syncytia, neuronal activity and the extracellular matrix^30–34^ as well as on the OPC-rich adjacent CC. In this way we were able to investigate, within the same anatomical areas, both neurogenesis and oligodendrogenesis, as well as the behavior of two distinct pools of brain progenitors, pbNSPCs and OPCs.

## Results

### Numbers of proliferating cells and of neuroblasts, in the SEZ, are reduced in lab mice and show seasonal variation in pine voles

To assess the behavior of pbNSPCs of the SEZ, we calculated the density and the percentage of proliferating cells (immunopositive for PCNA), as well as the density and the percentage of neuroblasts (immunopositive for Dcx) at the dorsolateral horn of the SEZ, a hotspot of pbNSPCs^35^ and the site of convergence for neuroblasts and oligodendroblasts ahead of their outward migration via the rostral migratory stream^36^. All PCNA + cells were found to co-express the transcription factor Sox2; thus, they were all considered to be pbNSPCs (Fig. 1A–C,E).

Firstly, we compared lab and wild mice (all bearing the typical 2n = 40 karyotype) and we found that the density and the percentage of proliferating cells and the density of neuroblasts were significantly higher in wild mice (Fig. 1F–G, Suppl. Fig. 2A–E), indicating that lab rearing is correlated to restricted proliferation and generation of neuroblasts in the SEZ. To test more for the effect of habitat, we looked in the SEZ of the *Microtus thomasi*, a fossorial rodent that spends a significant fraction of its life in confined spaces and relies more on the use of olfaction^37^. The less investigated *Microtus thomasi’s* SEZ had a structure similar to that of the mouse and the rat niche, as has been shown in the *Microtus ochrogaster*^38^, apart from the detection of individual proliferating cells located in the striatum (Fig. 1E), deeper than the 30–50 μm distance from the ventricular wall in which all proliferating cells are found in mice and rats, respectively^39^. Notably, a clear dichotomy in PCNA + and Dcx + cell densities was observed in the samples we analysed. We looked at body weight (as an indicator of age), gender and the season of capture (autumn versus spring), as factors possibly separating the two sub-populations and only seasons turned out to have a significant effect (Fig. 1F–G and data not shown). To ascertain appropriate comparisons, we also separated lab mice in spring and autumn pools and no seasonal variation was found in PCNA + and Dcx + cells. In voles, the SEZ was significantly richer in proliferating cells and neuroblasts in spring, with *Microtus*^*s*pring^ pbNSPCs exhibiting a behavior similar to that of Wild^2n=40(autumn)^ mice, while the *Microtus*^*autumn*^ population exhibited pbNSPC behavior similar to that of lab mice (the percentage of Dcx + cells was even significantly lower) (Fig. 1, Suppl. Fig. 2). Overall, these data reveal that the proliferative and neurogenic activity, per unit of SEZ, varies within a similar “low/high” range in mice and voles, and is directly affected by lab or wild habitat in mice and by seasons in the *Microtus*. It should be noted that the total number of PCNA + and Dcx + cells (across the full length of the SEZ) is expected to differ between species, based on species-specific differences in the size of the brain^39^.

### Numbers of proliferating cells and of neuroblasts in the SEZ are correlated to the number of chromosomes

**Fig. 2:**
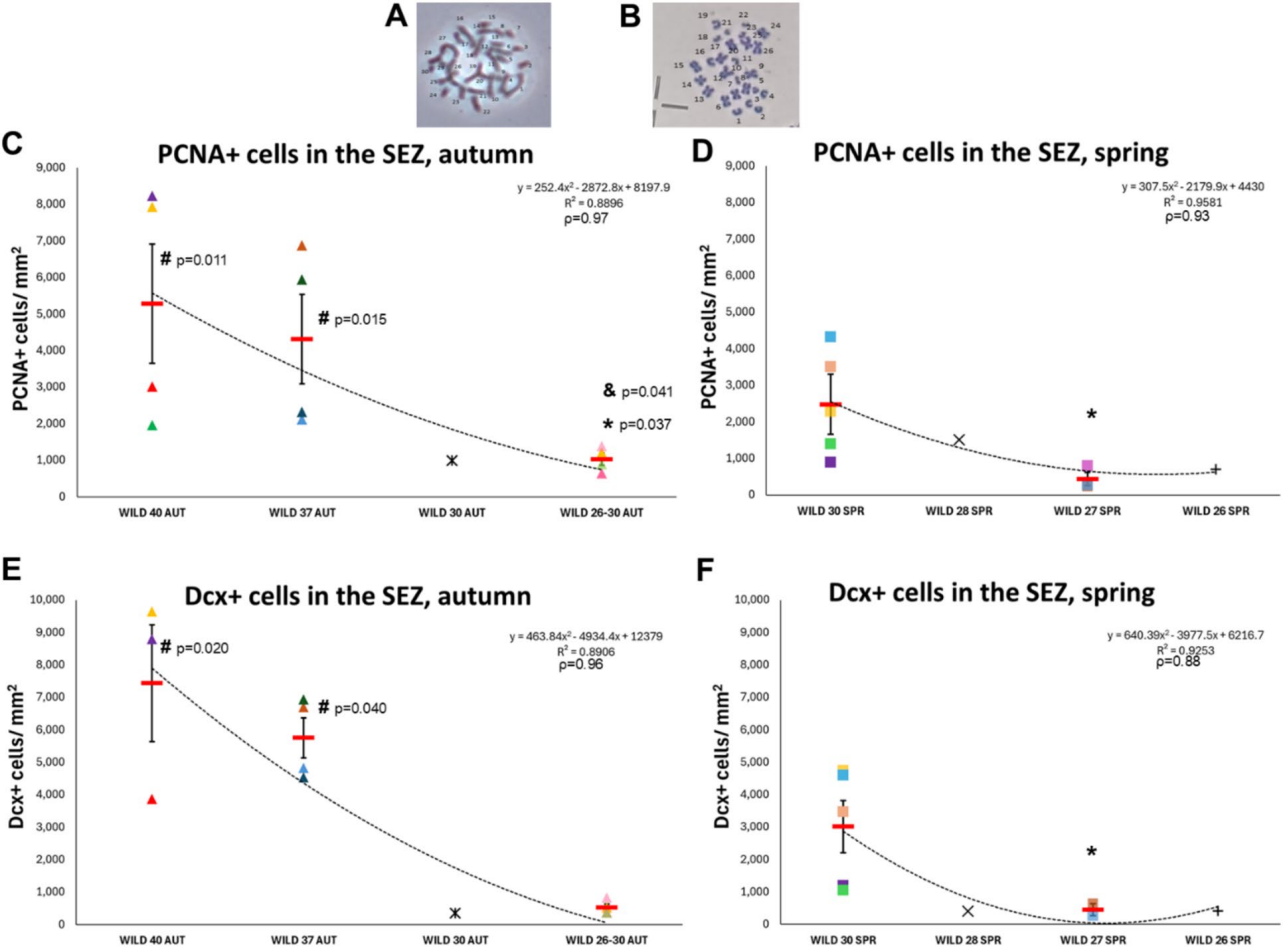
Effects of chromosomal fusions, leading to changes in chromosome numbers, on proliferation and neurogenesis in the SEZ niche. (**A**–**B**) High magnification images of *M. m. domesticus* metaphasic chromosomes from Wild^2n=40^ (in (**A**), without G-band staining) and Wild^2n=30^ (in (**B**), with G-band staining) individuals. (**C**–**F**) Scatter plot graphs showing the density of PCNA + and Dcx + cells within the SEZ of wild mice populations with different numbers of chromosomes, separately for autumn (**C**, **E**) and spring (**D**, **F**) collections. [Individual values are shown with different colours and shapes, as explained in Suppl. Fig. 1. Mean values are shown with red lines and error bars depict the SEM. Comparisons with lab mice (#) or with Wild^2n=40^ (&) or with Wild^2n=37^ (*) were performed using one-way ANOVA followed by Tukey’s *post-hoc* analysis. The polynomial equation describing each plot, the R-squared value, as well as the Pearson’s correlation (ρ) are shown at the upper right of each graph.]

Based on previous work^22^ and on explorative new collections, we sampled wild mice populations with atypical karyotypes, from different sites in Greece. Besides including wild mice with 2n = 40, we also worked with wild mice with 2n = 37, 30, 28, 27 and 26 chromosomes (Suppl. Table 1 and Suppl. Fig. 1, Fig. 2A,B). From one site (#7, in Mavroneri, Suppl. Fig. 1) we were not able to karyotype all mice, but as we have established well that mice found there belong to populations with 2n ≤ 30 (Suppl. Table 1), we grouped them as a separate Wild^2n≤30^ pool. When the densities and percentages of PCNA + and Dcx + cells were plotted against the number of chromosomes a surprising pattern was revealed, with all variables found to decrease in proportion to the reduction in the numbers of chromosomes (Fig. 2C–F, Suppl. Fig. 2C–F). The correlation was tested separately for mice captured in spring and autumn and was found to be very strong, with Pearson’s correlation values higher than 0.90. One-way ANOVA analyses revealed that, besides Wild^2n=40^ mice, Wild^2n=37^ mice had also significantly higher densities of PCNA + and Dcx + cells in the SEZ when compared to lab mice (Fig. 2C–F, Suppl. Fig. 2F–I). Wild mice with karyotypes 2n ≤ 30 showed no differences compared to lab mice and had significantly less PCNA + and Dcx + cells than Wild^2n=40^ and Wild^2n=37^ mice. The season was not found to affect the densities or percentages of PCNA + and Dcx + cells in wild mice (*p* > 0.05, *post-hoc* analyses for all-karyotype Wild^autumn^ versus all-karyotype Wild^spring^ mice as well as for Wild^2n≤30(autumn)^ versus Wild^2n≤30(spring)^), as was the case for lab mice.

### Oligodendrocyte progenitor cells are not affected by ecological factors and by changes in chromosome numbers

**Fig. 3:**
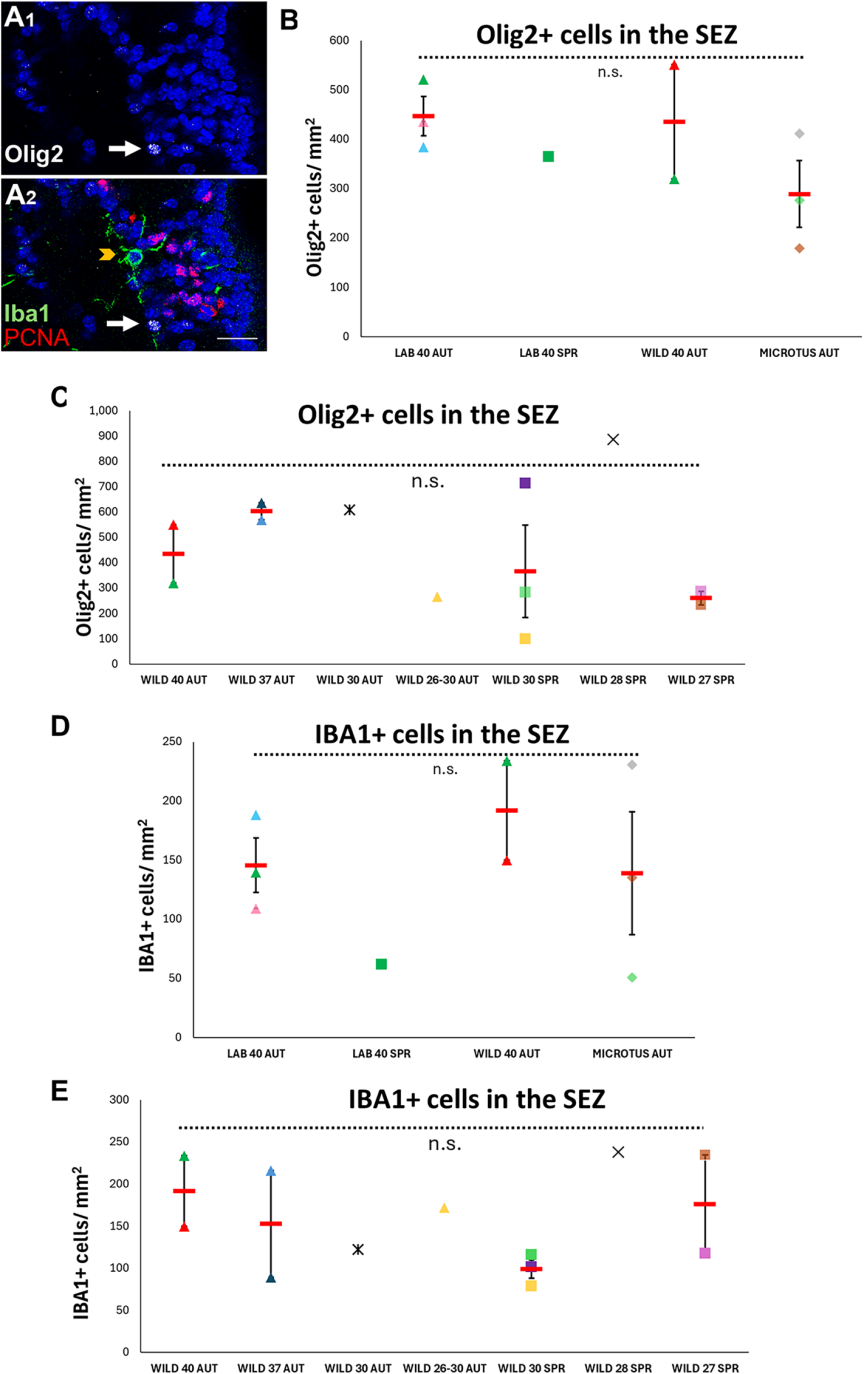
Effects of habitat, species, seasons and of chromosome numbers on the density of oligodendroblasts and microglial cells in the SEZ. (**A**) Detail of the “dorsal horn” of the SEZ in a Wild^2n=40^ mouse, with a typical Iba1 + microglial cell (in green, indicated by a yellow arrowhead, A2) and an Olig2 + cell (in white, indicated by a white arrow, A1). PCNA nuclear staining is in red. [scale bar: 15 μm] (**B**–**E**) Scatter plot graphs showing the density of Olig2 + (oligodendroblasts) and Iba1 + (microglia) cells within the SEZ of lab mice, wild mice populations with different numbers of chromosomes and *Microtus thomasi*. [Individual values are shown with different colours and shapes, as explained in Suppl. Fig. 1. Mean values are shown with red lines and error bars depict the SEM. Comparisons were performed using one-way ANOVA followed by Tukey’s *post-hoc* analysis. “n.s.”: not significant].

**Fig. 4:**
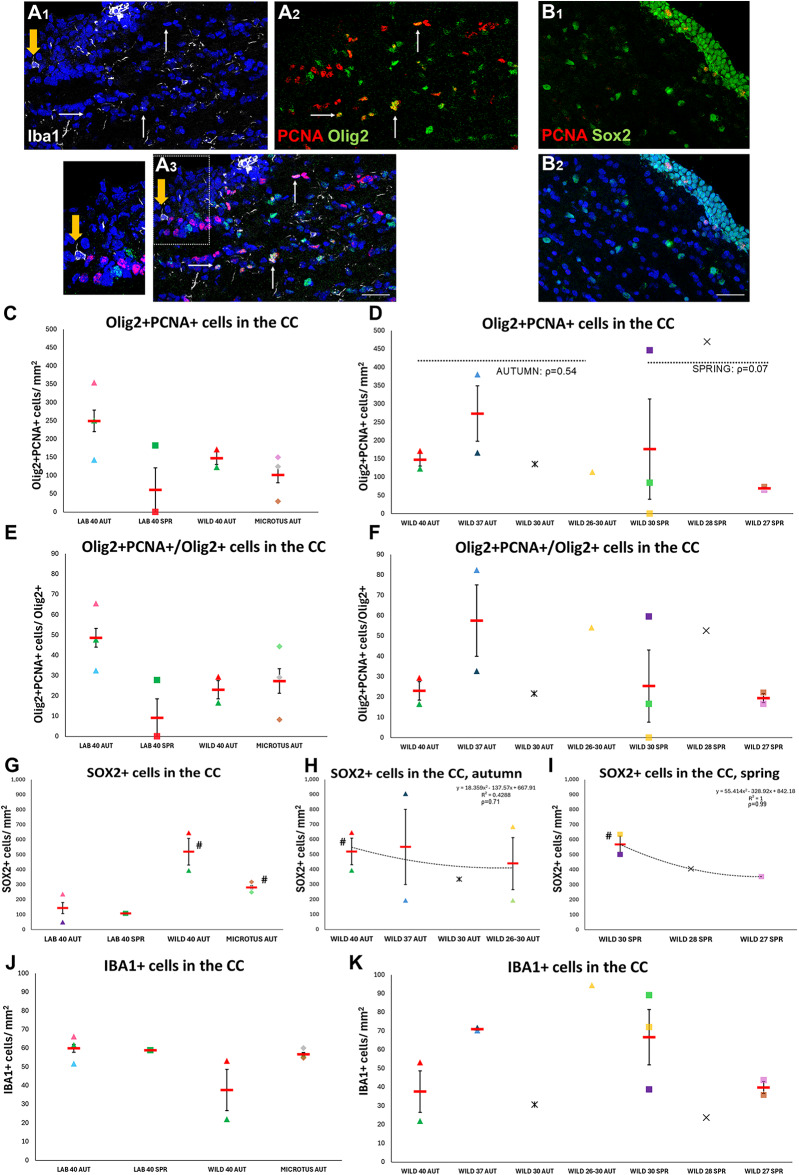
Effects of habitat, species, seasons and of chromosome numbers on the density of different cell types in the CC. (**A**) Image of the supraventricular CC with cells immunopositive for Iba1 (in white; a typical example is indicated by the yellow arrow with the area outlined in A3 shown in higher magnification), PCNA (in red) and Olig2 (in green). The analysis was focused on proliferating Olig2 + cells (examples indicated by white arrows). (**B**) Image of the supraventricular CC with cells immunopositive for Sox2 (in green; note the numerous immunopositive ependymal cells at the ventricular wall and the typical cytoarchitecture of the CC, with chains of nuclei) and PCNA (in red). [scale bar: 50 μm in A; 40 μm in **B**] (**C**–**K**) Scatter plot graphs showing the density of Olig2 + PCNA + double positive cells (**C**, **D**), the percentage of mitotic Olig2 + cells within the pool of Olig2 + cells (**E**, **F**), the density of Sox2 + cells (**G**–**I**) and of Iba1 + cells (**J**, **K**). [Individual values are shown with different colours and shapes, as explained in Suppl. Fig. 1. Mean values are shown with red lines and error bars depict the SEM. Comparisons to “lab mice” (#) were performed using one-way ANOVA followed by Tukey’s *post-hoc* analysis. The polynomial equation describing the plots, the R-squared value, as well as the Pearson’s correlation (ρ) are shown at the upper right in (**G**–**I**) and only the Pearson’s correlation (ρ) in (**D**)].

To assess if the effects we identified on the neurogenic output of the SEZ were also visible to its oligodendrogenic output, we calculated the density of Olig2 + oligodendroblasts in the dorsoventral horn of the niche (Fig. 3A–C). Moreover, we extended our analysis to the supraventricular CC, populated by OPCs of embryonic/early postnatal origin that self-renew and dynamically retain their density during homeostasis and demyelination^8,11^. Because in the CC Olig2 is expressed both in OPCs and in mature oligodendrocytes, we focused on the mitotic fraction of OPCs, i.e. the cells co-expressing Olig2 and PCNA (Fig. 4A–F). We also counted Sox2 + cells, as the expression of this transcription factor is undetectable in mature cells of the nervous system, but is retained in cells with neural progenitor identity of the brain parenchyma^40^ (Fig. 4G–I). Our analysis revealed that the density of Olig2 + cells in the SEZ and of mitotic Olig2 + cells in the CC were unaffected by species, habitat, or karyotype in the same groups that showed differences in PCNA + and Dcx + cells (Figs. 3, 4). On the other hand, the density of Sox2-expressing cells in the CC was significantly decreased in lab mice, compared to wild mice and to the *Microtus*^autumn^ (Fig. 4G–I). A strong Pearson’s correlation between the decreasing densities of Sox2 + cells and the decreasing number of chromosomes was apparent in the CC, as was observed for PCNA + and Dcx + cells in the SEZ (Fig. 4H–I).

### No differences in the behavior of pbNSPCs across experimental groups in vitro

**Fig. 5:**
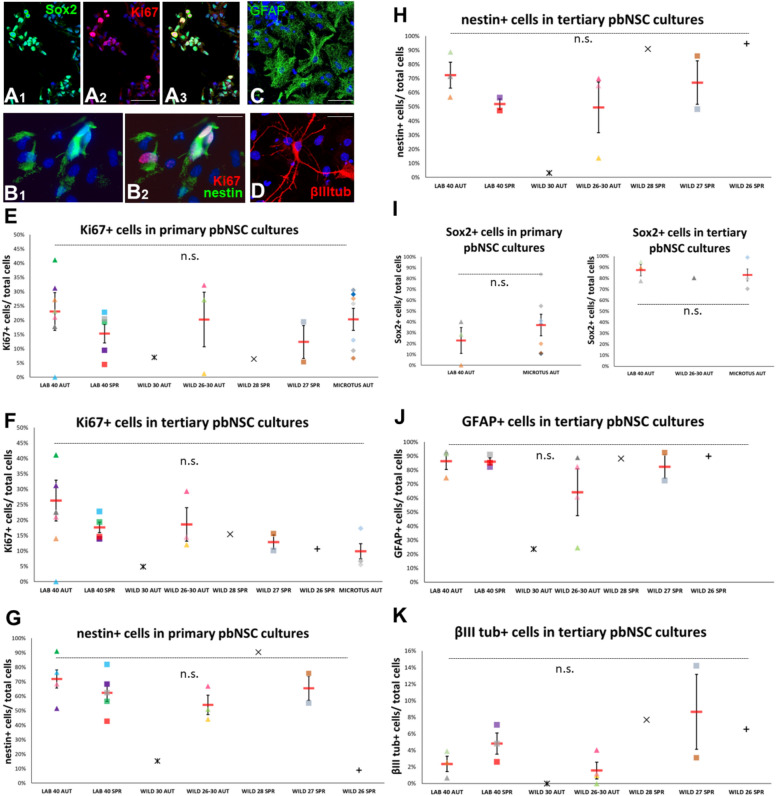
In vitro behavior of SEZ pbNSPCs. (**A**–**D**) Images of pbNSCs, immunostained for different cell-type markers. Expression of Sox2 (A1, in green) and Ki67 (A2, in red) in *Microtus*-derived primary neurosphere cells. (**B**) Expression of nestin (B1, in green) and Ki67 (B2, in red) in lab mouse-derived primary neurosphere cells. (**C**) Expression of βΙΙΙ-tubulin (in red) in Wild^2n=30^ mouse-derived tertiary neurosphere cells. (**D**) Expression of GFAP (in green) in Wild^2n=30^ mouse-derived tertiary neurosphere cells. [scale bars: 40 μm in (**A**, **C**); 10 μm in B; 15 μm in (**D**)] (**E**–**K**) Scatter plot graphs showing the percentages of Ki67 + cells (in **E**, **F**), of Nestin + cells (in **G**, **H**), of Sox2 + cells (in **I**), of GFAP + cells (in J), and of βIII tubulin + cells (in K) in primary and tertiary cultures of pbNSCs isolated from mice or *Microtus* (in **I**). [Individual values are shown with different colours and shapes, as explained in Suppl. Fig. 1. Mean values are shown with red lines and error bars depict the SEM. Comparisons were performed using one-way ANOVA followed by Tukey’s *post-hoc* analysis. “n.s.”: not significant].

The significant differences in the activity of SEZ pbNSPCs in animals living in diverse habitats, but also in mice harboring chromosomal fusions, could be caused by hard-wired, cell autonomous, differences, or by changes in external factors regulating their behavior. To test this in the most direct way, in some animals one brain hemisphere was included in the histological analyses, while the other was used for the isolation of SEZ-derived pbNSPCs and their culture in the form of free-floating colonies known as neurospheres. One-week old primary, or twice passaged (tertiary), neurospheres were dissociated and cells were plated on glass coverslips. After two days in pro-proliferation conditions, their mitotic activity (percentage of Ki67 + cells) and their NSPC profile (percentage of nestin + cells in mouse cultures and of Sox2 + cells in *Microtus* samples, because they failed to produce positive immunostaining for nestin) were assessed. In addition, tertiary mouse neurosphere cells were plated on glass coverslips and were analyzed after five days in pro-differentiation conditions for the presence of GFAP + astrocytes and βΙΙΙ-tubulin + neurons. Cell culture analyses revealed that pbNSPCs exhibited similar in vitro behavior irrespective of species, karyotype, season, and lifestyle (Fig. 5).

### Ecological and genetic factors do not alter the immediate NSPC cellular microenvironment, but the choroid plexus is bigger in wild animals

The absence of differences in the in vitro behavior of pbNSPCs strongly indicated that the effects observed in vivo are instructed by external factors. To assess if these changes were correlated to changes in the immediate cellular microenvironment of the SEZ, we calculated the pool of what we termed “supporting cells” of the SEZ, i.e. the cells of the niche that do not belong in the pbNSPC lineage, by subtracting from the total number of cells (all DAPI + nuclei) the numbers of all PCNA + and Dcx + cells. This pool of cells includes mainly ependymal and endothelial cells, as well as differentiated astrocytes^35,41^ and microglia^42,43^. Notably, we found that the population of supporting SEZ cells remained unaffected (Suppl. Fig. 3). We also looked at microglial (Iba1 +) cells that act as regulators of pbNSPCs^42–44^ and we found that their density remained stable across all animal groups in the SEZ and the CC (Figs. 3D–E, 4J–K). Because the number of microglial cells is not always sufficient to indicate a response of this cell population^45,46^, we also calculated their cell body size, across different animal groups and we counted the number of processes per microglial cell, again not finding any significant differences (Suppl. Fig. 4B and data not shown). Another element of the ventricular microenvironment that has been shown to regulate the activity of NSCs is the choroid plexus^32^, a floating epithelial-like structure stemming from the floor of the anterior lateral ventricles. We assessed the size of the choroid plexus by noting its presence along the rostro-caudal length of the lateral ventricles^47^ and we found that in lab mice, compared to wild animals, it was restricted to more caudal parts of the lateral ventricles (Sup Fig. 4F,H) and it was less dense and smaller (Suppl. Fig. 4G,H).

## Discussion

The behavior (e.g. proliferation, differentiation, survival/maturation) and regulation of pbNSPCs, especially in their niches, has been investigated extensively in mice maintained in animal facilities, under tightly controlled conditions that differ substantially from the natural habitats of these rodents. Accumulating evidence suggests that environmental enrichment^17,20^, increased physical activity^14,48^ and high social interactions^16^ are correlated with increased levels of neurogenesis, mainly in the hippocampal niche. Moreover, a large comparative analysis of hippocampal neurogenesis across different European and southern African Muridae species revealed significant differences, reinforcing the hypothesis that neurogenesis shows signs of adaptation to environmental conditions^49^. Further experimental work with wild mice revealed that in captured wood mice and Western house mice, an increase in physical activity did not lead to increased neurogenesis^50,51^, in contrast to what has been widely observed in lab mice^14,48,51^. Finally, mice domestication^52^, as well as their rearing in different animal facilities^53^ has been shown to affect phenotypic traits, even at the level of chromatin organization. Based on the above, we considered wild mice as our control population and we hypothesized that the behavior of pbNSPCs in lab mice could be critically different. We decided to investigate the SEZ stem cell niche that is located and structured in a way that enables the integration of signals originating from multiple pathways^4,31,32,34^ and contributes to olfaction. Indeed, our data revealed that proliferation and neurogenesis in SEZ pbNSPCs are affected by life conditions and were decreased (by approximately 50–70%) in lab mice when compared to wild animals. In agreement with the observations on hippocampal neurogenesis in wild mice, this sets a new, significantly higher, translational “standard” when interpreting the therapeutic potential of experimental manipulations that lead to increased levels of neurogenesis, with the behavior of pbNSPCs in wild mice offering a “real mouse world” comparator.

Wild mice provide a unique opportunity to generate valid, unbiased from experimental handling, information, especially because even mild interventions can affect the mouse characteristics^52^ or the vole’s neurogenic activity^38^. To achieve this, it is essential to identify multiple, well-defined, wild populations that will allow the extraction of key conclusions by linking genetic and molecular data with descriptive data, as has been done with human samples^54–56^. Here, we identify interesting comparator groups either at the cellular or at the animal level. At the cellular level, the divergent behavior of neuronal and of oligodendroglial lineage progenitors most probably underlines the divergent roles and evolutionary adaptations of the two cell lineages. The former have acquired a dependence on the niche microenvironment wherein their activity is tightly regulated^1,57–59^ and the latter have adapted to life in the parenchyma^60,61^ exhibiting persistent generation of myelin^11^; albeit, remain responsive to life-style changes, such as piano playing^62,63^. In contrast, Sox2 + neural progenitors located in the CC showed behavior similar to that of neural progenitors in the SEZ. The identification of cell pools with convergent/divergent aspects of behavior in the same brain areas can enrich the analytical resolution of methods incorporating anatomical elements (such as spatial transcriptomics).

At the populations level, we show that the density of pbNSPCs in the SEZ exhibits seasonal variation in the *Microtus*, but not in mice, an observation that can be used to extract key metabolic information by identifying systemic factors with similar seasonal fluctuations. This discrepancy could reflect the fact that wild mice populate humanized habitats, characterized by higher stability, throughout the year, in terms of temperature, abundance of food and the presence of predators, while the *Microtus’* ethology involves seasonal variation in physical, reproductive and food scavenging activity. Because two of the three voles captured in spring had lower body weight, indicating younger age, when also checked for a possible correlation between body weight and density of PCNA + cells, this turned to be negative (Pearson’s analysis ρ = − 0.43), while a t-test for the density of PCNA + cells in individuals with body weight < 10 g versus body weight > 20 g gave no significant result (*p* = 0.09). It should be noted that, overall, we found no significant differences in the gross cyto-architecture of the SEZ between lab mice, wild mice and voles and the same was observed when comparing mice and rats^39^. This suggests that valid comparisons can be drawn by investigating species that belong to the same order (rodentia) and that differences in the behavior and organization of postnatal brain progenitors require longer phylogenetic distances^64^.

Importantly, the observation that the differences identified between lab and wild rodents are not hard-wired and cell-autonomous, because they are not observed in vitro, directs the focus towards metabolic/systemic targets. Since differences observed in vivo were very strong and because of the limited number of wild animal samples, the in vitro assays we employed were purposely crude (e.g. cells were grown in abundance of EGF and FGF2, or in the total absence of these factors). Additional work will be necessary to assess more conclusively the numbers of neural stem cells in the parent SEZ, or the detailed response of cells to different factors^65^. Accumulating evidence has shown that circulation^48,66^ and choroid plexus-derived^32,33,67^ factors are important in the control of pbNSPCs and our data offer a unique opportunity to expand such analyses using “real world” data (e.g. metabolic and inflammatory profiles). An interesting observation was that of a smaller choroid plexus in lab animals, restricted in the more caudal parts of the lateral ventricles, where the pbNSC density is lower^35^. Nevertheless, the size of the plexus did not directly correlate with the levels of pbNSC activity in the different wild populations. This could be because even more important than the size is the choroid plexus secretome which, for example, in lab mice switches from promoting to restricting pbNSC activity over ageing^32^. It could also indicate that the plexus size reflects the effects of other environmental factors, such as viral infections^68^, a finding also relevant to the strong trend of a decrease in the cell body of microglial cells in wild animals.

The most unexpected observation was the strong correlation between the decrease in the number of chromosomes and the decrease in the density of pbNSPCs in the SEZ. A mechanistical explanation for this will require additional analyses, such as G-banded chromosome staining, or some type of NGS and ATAC sequence analyses, which can now be performed on archived material. Gene silencing due to “position effect variegation” has been described in mice^69^; however, rather than focusing on individual genes, the involvement of pericentromeric and of telomeric chromatin seems to offer a more valid target. These are the chromosome areas quantitively affected by Rb and they have been implicated in the control of stem cell proliferation^70,71^ and of the age-related decline in stem cell function^72,73^.

In terms of basic biology, our data reveal a key feature of the regulation of pbNSPCs and of the SEZ function in rodents. The fact that the *Microtus* niche becomes almost void of progenitors in the autumn, only to be replenished in spring, indicates that living conditions affect the proliferative activity of pbNSPCs, possibly acting at the top of the hierarchy, at the quiescence-to-mitotic activation transition of neural stem cells^2^. At the same time, the fact that the density of supporting niche cells (ependyma, endothelium) remains similar across species, karyotypes, lifestyle (even in the lab animals that experience many generations of hypoactivity), and seasons, strongly suggests that the structure of the niche is independent of any fluctuations of neurogenic activity. These findings might reconcile previous observations on the human SEZ, that has been reported to become depleted of pbNSPC activity during infancy^58,59,74^, but also to be able to respond in cases of degeneration, even in the elderly^75,76^. Further histological analysis of niche elements, such as the vasculature, will consolidate this architectural stability and can lead to the calculation of a “maximum capacity” for pbNSPC density per niche unit.

One methodological limitation of this study is that the age of wild animals could not be reliably determined. The only animal groups with certified ages were the laboratory mice ranging from 2 to 4 months. We could only be sure that wild mice were adult (post-1 month old), based on their body size, and that the females were not pregnant, a condition recently reported to affect neurogenesis in the SEZ^12^. Neurogenesis (and more widely the activity of tissue-specific stem cells) decreases over ageing in lab animals^77,78^. Therefore, lab animals included in the study were at the peak of neurogenesis age, while wild mice were not younger than lab mice, rather a mixture of different ages (old, or sick wild animals are not very active; thus, are more rarely captured and are under-represented). In conclusion, the overall results indicate that it is highly unlikely that the age of wild mice might have confounded our results, as the activity of pbNSPCs was found to be both increased and decreased (compared to the age-grouped young adult lab mice) in different groups of wild mice; whilst at the same time, microglial cells and OPCs showed different patterns of behavior.

Another possible confounding factor in this type of work is the possible lack of genetic variation, if individuals captured at one site belong to the same family. In this study there was no implementation of strict distribution rules, such as the use of grids employed in field work destined for the assessment of the diversity of fauna and flora, and all captured individuals were included. Supplemental Table 1 and supplemental Fig. 1 provide details that inform regarding possible family relations between individuals. The 4 Wild^2n=40^ mice were captured at 4 different locations; thus, have no family relations to each other. The 4 Wild^2n=37^ mice were captured at the same location and time. Nevertheless, our analysis revealed a wide range of values, especially in the density of PCNA + and Dcx + cells in their SEZs, whilst the grouping of R586 and R599 observed in the density of PCNA + , Dcx + and Olig2 + cells is not repeated when looking at the density of Iba1 + cells. The 6 Wild^2n=30^ mice were captured as two groups: R613, R614, R617 and R619 in location #5 and Wild4, Wild6 in the very distant location #7. Wild6 measurements do not show segregation from the other individuals of the same group (Wild^2n=30, spring^). Furthermore, Wild4 and Wild6 show: (a) similar patterns in the density of PCNA + and Dcx + cells in the SEZ, (b) moderately different patterns in the density of Olig2 + and Iba1 + cells in the SEZ, (c) strongly different patterns in nestin +, GFAP + and βIIItubulin + cells in cultures. The other 12 wild mice were also captured at Mavroneri (location #7 in the map) in the same barn and were trapped at the same time, apart from Wild13 that was captured almost a year later. There is no direct evidence on family relations amongst the individuals that share the same numbers of chromosomes, but the co-existence of individuals with different karyotypes strongly indicates fluidity of populations. Voles were captured in two, distant, areas. The species lives in highly complicated burrows, that can extend to more than 25 m in total length^79^. Each network is usually inhabited by one male/female pair, although coexistence of additional adults has been reported and social exposure has been shown to affect neurogenesis in *Microtus ochrogaster*^65^. Only one adult was captured per burrow; thus, limiting the possibility of 1st or 2nd degree relations.

## Conclusions

The worth of field-work expeditions to capture wild mice, rats and voles and the benefit of gathering descriptive information, in the absence of experimental manipulation, depends on two conditions: (a) choice of the appropriate comparators and (b) availability of high throughput methods to extract information. As large-scale genomic, proteomic, metabolomic and imaging technologies are becoming available, the first condition becomes even more significant. It is similar to the human setting. The generation of single-cell analysis genomic data from specialized populations, such as the age-resistant supercentenarians^80^, astronauts exposed to extreme gravitation conditions^55^ or multiple sclerosis patients of different disease stages^56,81^ provided novel information on the biological processes of ageing, of the effects of space and of disease. Such investigations are empowered by clustering individuals that belong in large-scale pools using technologies such as multimodal brain imaging, cognitive tests or a range of biochemical analyses^82^. Here, we identified rodent populations (wild, captured at different seasons, living in humanized habitats) with significantly divergent behavior of pbNSPCs (phenotype). The application of proteomic or metabolomic analyses will enable the identification of the factors that mediate the phenotypic differences. Notably, the strong correlation between phenotype and the number of chromosome fusions that can only be explained by proportional changes in chromatin structure (genetic or epigenetic, possibly affecting pericentromeric chromatin), should be feasible to investigate further using advanced sequencing technology.

## Materials and Methods

### Animals, euthanasia method and bone marrow karyotyping

Lab mice were of the Bl6CBAC and of the 129sv backgrounds. All animals were between 2 and 4 months of age, inbred at the designated animal facility of the University of Patras (EL13 BIOexp-04), maintained in steady light/dark cycle (12/12 h) with free access to food and water. Animal breeding, maintenance and handling was performed in accordance with the European Communities Council Directive Guidelines (86/609/EEC) for the care and use of Laboratory animals as implemented in Greece by the Presidential Decree 56/2013 and approved and scrutinized by the West of Greece Prefectural Animal Care and Use Committee (Protocol number: 118188/432/21-05-2020). Wild mice (*Mus musculus domesticus*) were trapped alive with Sherman traps in a variety of humanized habitats, e.g. storage buildings and farms. Thomas’s pine voles (*Microtus thomasi*) were trapped alive with wire custom-made, rectangular traps of 5 × 6 × 20 cm size. Bait (usually pieces of carrot) was put deep in the trap, with an additional piece positioned at the opening, under a shutter. The traps were set, fully aligned and at the same depth, at the open ends of tunnels, to resemble an extension of the burrow. The underground burrows were located in cultivated and uncultivated fields. Both taxa are common in nature and not under threat or protection by national or international decrees. All field procedures involving these wild animals were in compliance with guidelines, approved by the American Society of Mammalogists (Sikes and Gannon, 2011). The captured animals were brought to the lab and were maintained for the minimal possible duration (typically 2 to 7 days, with the exception of the wild^2n=30–26^ subgroup that was analyzed in spring) under standard animal house conditions and free access to water and food. All animals were subjected to colchicine pretreatment (0.025%, 2 μl/g of body weigh) 45 min prior to euthanasia.

Euthanasia was performed by final (non-recovering) anesthesia, with ketamine overdose and subsequent intracardially perfusion with fresh, ice-cold saline (50 ml per animal).

For karyotyping using bone marrow cells, chromosome preparations were obtained using a modified version of the direct bone marrow method^83^, incubating cell preparations with a hypotonic solution of KCl (0.75 M; 30 min at 37 °C), followed by fixation in Carnoy’s solution (3:1 methanol : glacial acetic acid). Slides were prepared by the standard air-drying technique. Metaphasic chromosomes were photographed in brightfield using the 100X objective of an Axioskop 2 Plus Zeiss microscope and a Zeiss mrc5 digital camera. Numbers of chromosomes were counted, in at least 3 metaphases. No animals were excluded and all protocols and analyses were planned and performed in compliance with the ARRIVE guidelines, as described in https://arriveguidelines.org/arrive-guidelines.

### Cell cultures, immunocytochemistry and pbNSPC karyotyping

For pbNSPC cultures, the subependymal zone of one brain hemisphere was dissected under the stereoscope using anatomical landmarks, as performed previously^5^ and was dissociated using accutase. Cells were plated in 6-well plates with proliferation medium (high glucose DMEM, FGF2 and EGF at 20 ng/ml, 2% B27 and 1% N2 supplements) and were allowed to grow as 3D free-floating aggregates called neurospheres. For the immunocytochemical analysis, the primary neurospheres were dissociated and plated on poly-D-lysine coated glass coverslips. Cells were fixed with 2% PFA after 48 h. Immunofluorescence was performed with standard protocols^5^, using the following primary antibodies: chicken anti-nestin (Abcam, 1/200, ab134017), mouse anti-nestin (Abcam, 1/200, ab6142), rabbit anti-Ki67 (Abcam, 1/500, [SP6] ab16667), goat anti-GFAP (Abcam, 1/700, ab53554), goat anti-Sox2 (Santa Cruz, 1/200, sc-17320), mouse anti-βΙΙΙ tubulin (Abcam, 1/500, Abcam, ab7751). Appropriate secondary antibodies (Biotium) were used, and nuclei were counterstained with Dapi (Merck). For karyotyping, neurosphere cells, of higher than two passages, were dissociated. After 48 h they were dissociated again, cells were incubated for 8.5 h with 10 μg/ml colchicine and were karyotyped with the bone marrow protocol, adjusted for pbNSPCs.

### Tissue handling for immunohistochemistry

After the intracardial perfusion with saline, the tissue was fixed in 4% PFA (4 °C, overnight), cryopreserved in 30% sucrose and frozen at − 80 °C. 14 μm-thick coronal cryo-sections were obtained (Leica) and were immunostained using standard protocols^5^, using the following antibodies: mouse anti-PCNA (Abcam, 1/500, [PC10] ab29), rabbit anti-Dcx (Abcam, 1/500, ab18723), goat anti-Iba1 (Abcam, 1/1000, AB5076), rabbit anti-Olig2 (Millipore, 1/300, AB9610), goat anti-Sox2 (Santa Cruz, 1/200, sc-17320). Appropriate secondary antibodies (Biotium) were used and nuclei were counterstained with Dapi (Merck).

### Imaging and cell count

Tissue sections and cells on coverslips were photographed with a Leica TCS SP8 confocal microscope, using the 40× objective and the Leica Application Suite × software. For SEZ measurements, at least two coronal sections, at different rostro-caudal levels (typically 1.2 and 0.8 mm from bregma) were imaged, with at least 2 optical fields at the “dorsal horn” domain of the niche (as shown in Fig. 1A). Cells were counted manually at a depth of 30 μm (mice and *Microtus*) or 50 μm (rats) from the ventricular wall; that we have previously shown to contain the SEZ^39^, as well as deeper in the initial segment of the rostral migratory stream. Cell densities are given as cells/mm^2^. For cell cultures, at least 10 optical fields were photographed per coverslip. It should be noted that nestin immunoreactivity could not be detected in *Microtus* cells, using two different antibodies (Abcam, chicken and mouse anti-nestin). For CC measurements, the same sections and methods were used, by photographing callosal areas adjacent to the lateral ventricles.

### Statistics

All cell counts were performed blind as to the experimental group and the karyotype and from multiple investigators. Statistical analyses were performed using IBM SPSS statistics (for 2-way and 1-way ANOVA, followed by the Tukey *post-hoc* analysis, whenever necessary). Scatter plots were generated, and regression and Pearson’s correlation analyses were performed using Microsoft Office Excel. All animals (irrespective of numbers of samples per population) were used for the correlation analyses, but only the populations with n ≥ 3 were used for ANOVAs.

## Supplementary Information

Supplementary Information.

## Data Availability

The datasets used and analysed during the current study are available from the corresponding authors on reasonable request.
