# Supplementary material for: Lab life, seasons and chromosome fusions affect non-cell-autonomously proliferation and neurogenesis, but not oligodendrogenesis, in mice and voles
Source: Sci Rep. 2025 May 28;15:18737. doi: 10.1038/s41598-025-01670-3 (PMC12119966; doi:10.1038/s41598-025-01670-3)
Supplement: Supplementary file 1 — Supplementary Information. [file 41598_2025_1670_MOESM1_ESM.pdf]

Supplemental Figures and Tables

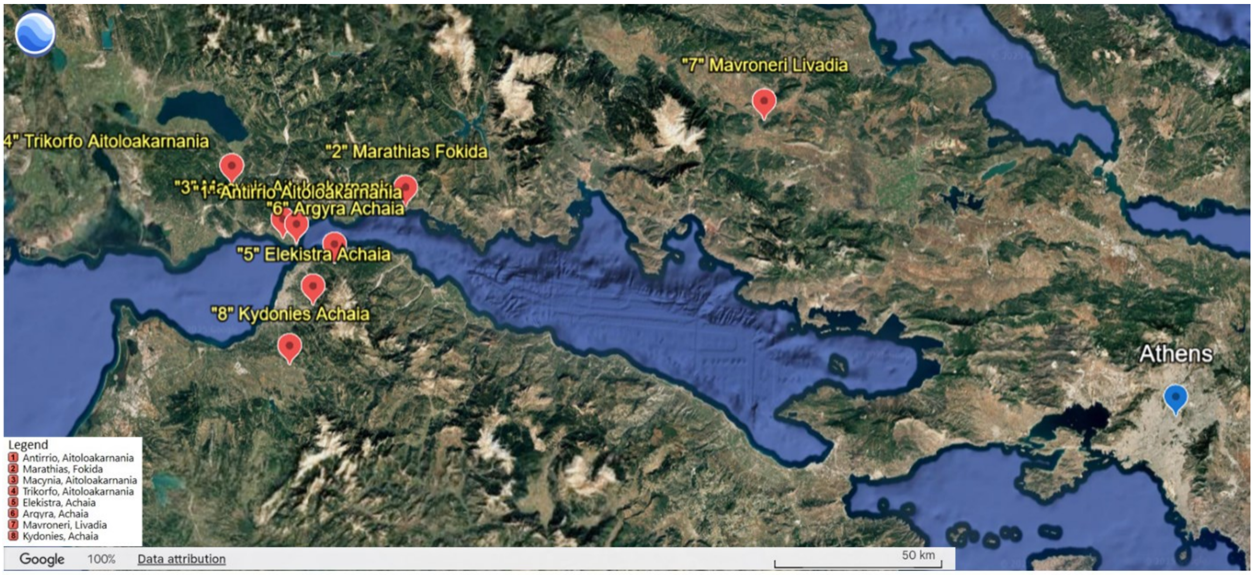

AUTUMN

Lab 40	Wild 40	Wild 37	Wild 30	Wild 26-30	Microtus
▲ E3	▲ R581	▲ R586	⁎ Wild 4	▲ Wild 1	◆ Microtus 1
▲ E5	▲ R582	▲ R599		▲ Wild 2	◆ Microtus 2
▲ E4	▲ R583	▲ R597		▲ Wild 3	◆ Microtus 3
▲ E8	▲ R584	▲ R600		▲ Wild 5	◆ Microtus 4
▲ Lab15				▲ Wild13	◆ Microtus 5
▲ Lab16					◆ Microtus 6
▲ Lab17					◆ Microtus 7
▲ Lab18					◆ Microtus 8
▲ Lab29					◆ Microtus 9
▲ Lab30					
▲ Lab31					

SPRING

Lab 40	Wild 30	Wild 28	Wild 27	Wild 26	Microtus
■ 3	■ R614	× Wild 8	■ Wild7	+ Wild 11	◆ 625
■ E7	■ R617		■ Widl9		◆ 626
■ M	■ R619		■ Wild10		◆ 627
■ Lab37	■ R613				
■ Lab39	■ Wild6				
■ Lab40					
■ Lab41					
■ Lab42					

Supplemental Figure 1. Details of wild animal collections

(Upper panel) Map of central Greece and northern Peloponnese, showing the collection areas of the different populations of wild animals. *Mus musculus domesticus* individuals, of different karyotypes, were captured at 6 different sites and *Microtus thomasi* at 2 sites. (1) Antirrio, Aitoloakarnania. (2) Marathias, Fokida. (3) Macynia, Altoloakarnania. (4) Trikorfo, Aitoloakarnania. (5) Elekistra, Achaia. (6) Argyra, Achaia. (7) Mavroneri, Voiotia. (8) Kydonies, Achaia. (Lower panel) Legend of the identity tag, of the gender (for female animals) and of the colour/shape with which each animal's measurements are depicted in the scatter plots. See also Supplemental Table 1. [The map was constructed in Google Earth and includes data from: Google, Data SIO, NOAA, U.S. Navy, NGA, GEBCO, Landsat/Copernicus, from the dates:14/12/2015–01/01/2021. It is used under the principles of fair use as described in Google Earth's "Terms of Service", available at https://www.google.com/help/terms_maps/)]

17

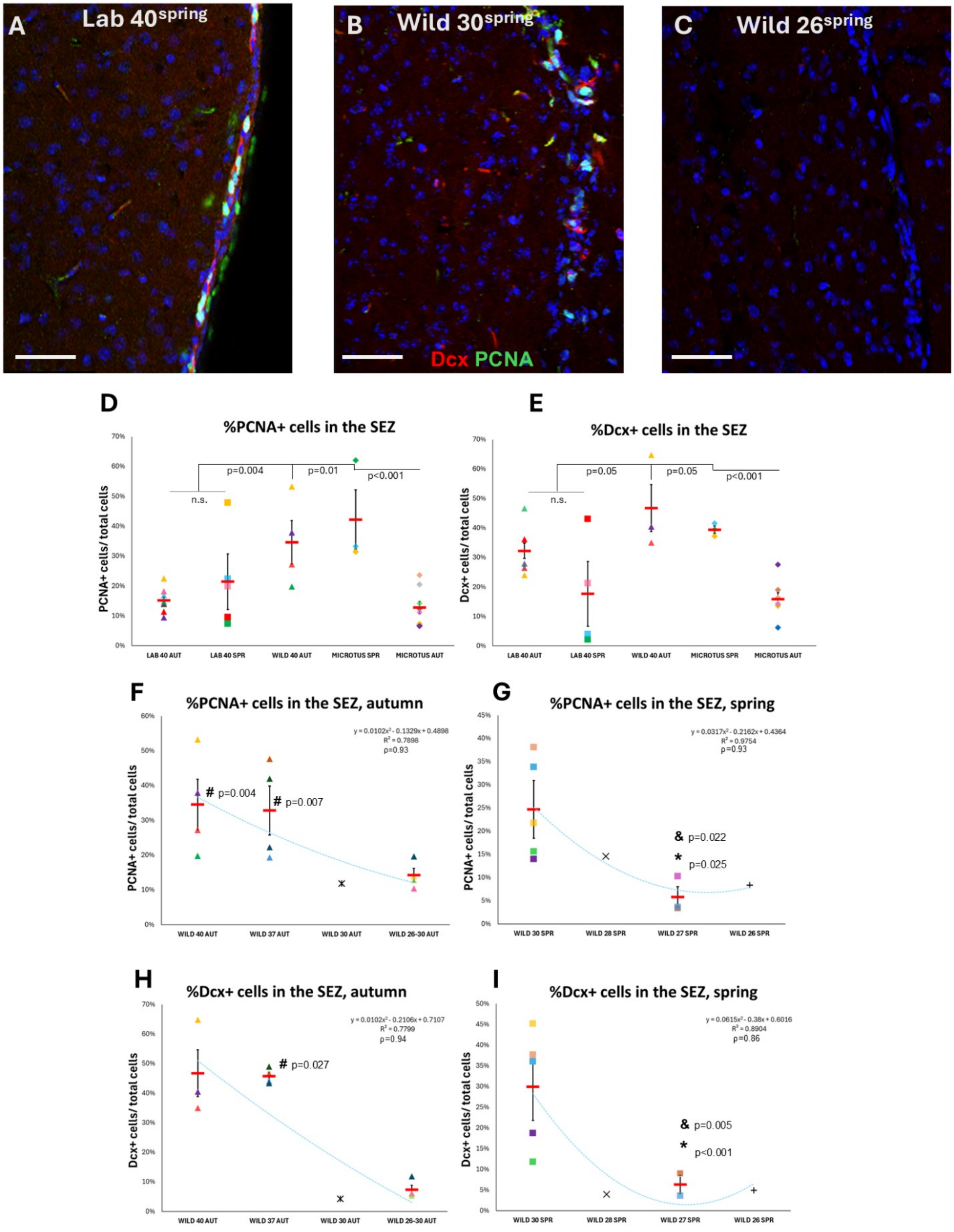

A Lab 40spring
B Wild 30spring
C Wild 26spring
Dcx PCNA
D
%PCNA+ cells in the SEZ
PCNA+ cells/ total cells
n.s.
p=0.004
p=0.01
p<0.001
LAB 40 AUT
LAB 40 SPR
WILD 40 AUT
MICROTUS SPR
MICROTUS AUT
E
%Dcx+ cells in the SEZ
Dcx+ cells/ total cells
n.s.
p=0.05
p=0.05
p<0.001
F
%PCNA+ cells in the SEZ, autumn
y = 0.0102x² - 0.1329x + 0.4898
R² = 0.7898
ρ=0.93
# p=0.004
# p=0.007
WILD 40 AUT
WILD 37 AUT
WILD 30 AUT
WILD 26-30 AUT
G
%PCNA+ cells in the SEZ, spring
y = 0.0317x² - 0.2162x + 0.4364
R² = 0.9754
ρ=0.93
& p=0.022
* p=0.025
WILD 30 SPR
WILD 28 SPR
WILD 27 SPR
WILD 26 SPR
H
%Dcx+ cells in the SEZ, autumn
y = 0.0102x² - 0.2106x + 0.7107
R² = 0.7799
ρ=0.94
# p=0.027
I
%Dcx+ cells in the SEZ, spring
y = 0.0615x² - 0.38x + 0.6016
R² = 0.8904
ρ=0.86
& p=0.005
* p<0.001

Supplemental Figure 2. Effects of habitat, species, seasons and of chromosome numbers on the percentages of proliferating cells and of neuroblasts in the SEZ

(A-C) Microphotographs of the SEZ in lab mice (A), Wild30 mice (B) and Wild26 mice (C), after immunostaining for PCNA (to mark proliferating cells, in green) and Dcx (to mark neuroblasts, in red). Note the stark reduction in the presence of PCNA+ and Dcx+ cells in the SEZ of Wild26 mice. [scale bars: 200μm]. (D,E) Scatter plot graphs showing the percentages of PCNA+ and Dcx+ cells within the SEZ of lab and wild rodent populations, at different seasons. (F,H) Graphs showing the percentages of PCNA+ and Dcx+ cells in wild mice captured in autumn, plotted against numbers of chromosomes. (G,I) Graphs showing the percentages of PCNA+ and Dcx+ cells in wild mice captured in spring, plotted against numbers of chromosomes. [Individual values are shown with different colours and shapes, as explained in Suppl. Figure 1. Mean values are shown with red lines and error bars depict the SEM. Comparisons with lab mice (#), with $Wild^{2n=40}$ (&) or $Wild^{2n=37}$ (*) were performed using one-way ANOVA followed by Tukey's *post-hoc* analysis. The polynomial equation describing each plot, the R-squared value, as well as the Pearson's correlation (ρ) are shown at the upper right of each graph.]

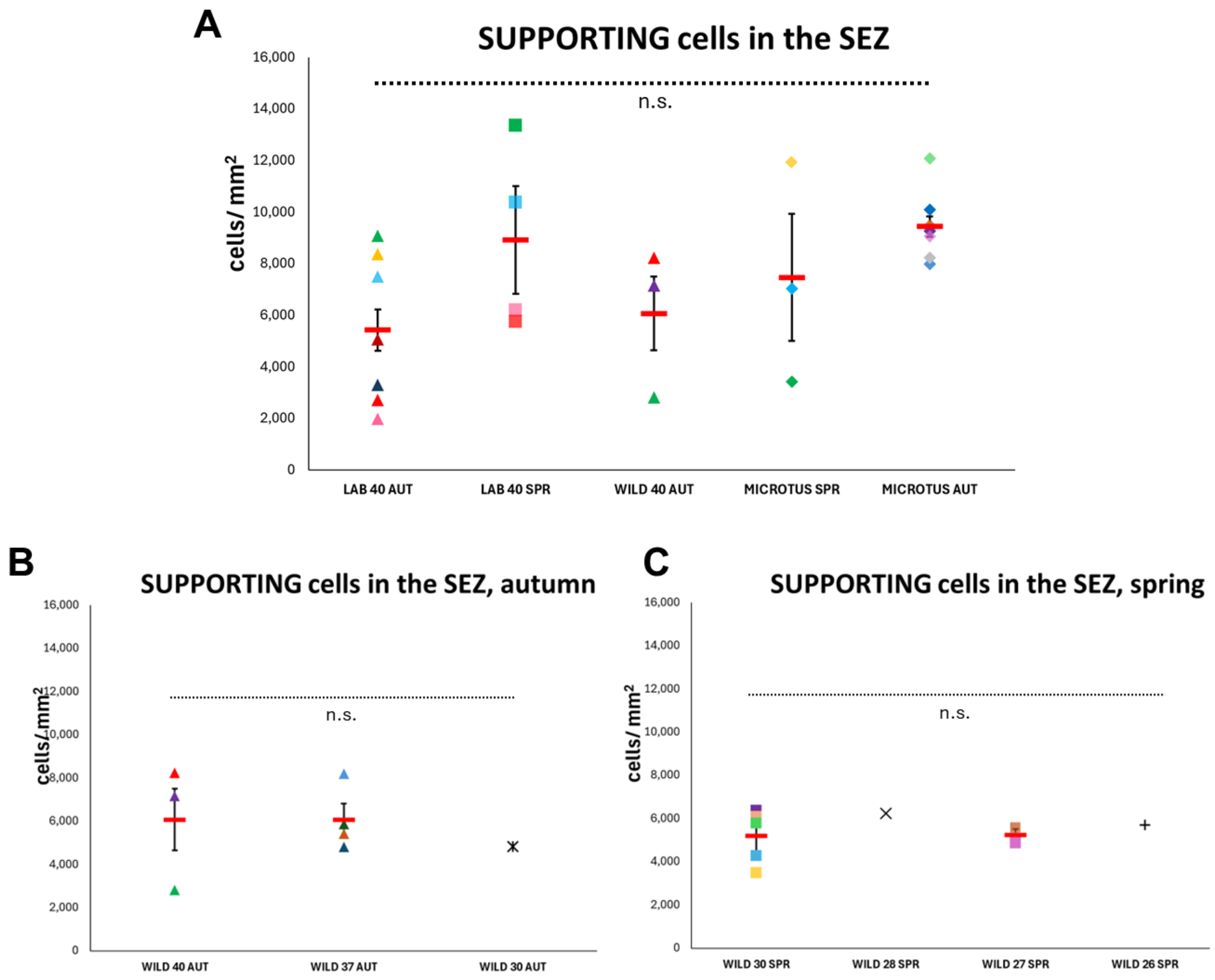
A
SUPPORTING cells in the SEZ
cells/ mm^2
16,000
14,000
12,000
10,000
8,000
6,000
4,000
2,000
0
n.s.
LAB 40 AUT
LAB 40 SPR
WILD 40 AUT
MICROTUS SPR
MICROTUS AUT
B
SUPPORTING cells in the SEZ, autumn
cells/ mm^2
n.s.
WILD 40 AUT
WILD 37 AUT
WILD 30 AUT
C
SUPPORTING cells in the SEZ, spring
cells/ mm^2
n.s.
WILD 30 SPR
WILD 28 SPR
WILD 27 SPR
WILD 26 SPR

Supplemental Figure 3. Effects of habitat, species, seasons and of chromosome numbers on the density of supporting cells in the SEZ

(A-C) Scatter plot graphs of the density of supporting cells within the SEZ of different rodent populations, according to life conditions (lab or wild), seasons, species and numbers of chromosomes. [Individual values are shown with different colours and shapes, as explained in Suppl. Figure 1. Mean values are shown with red lines and error bars depict the SEM. Comparisons were performed using one-way ANOVA followed by Tukey's *post-hoc* analysis. "n.s.": not significant]

46

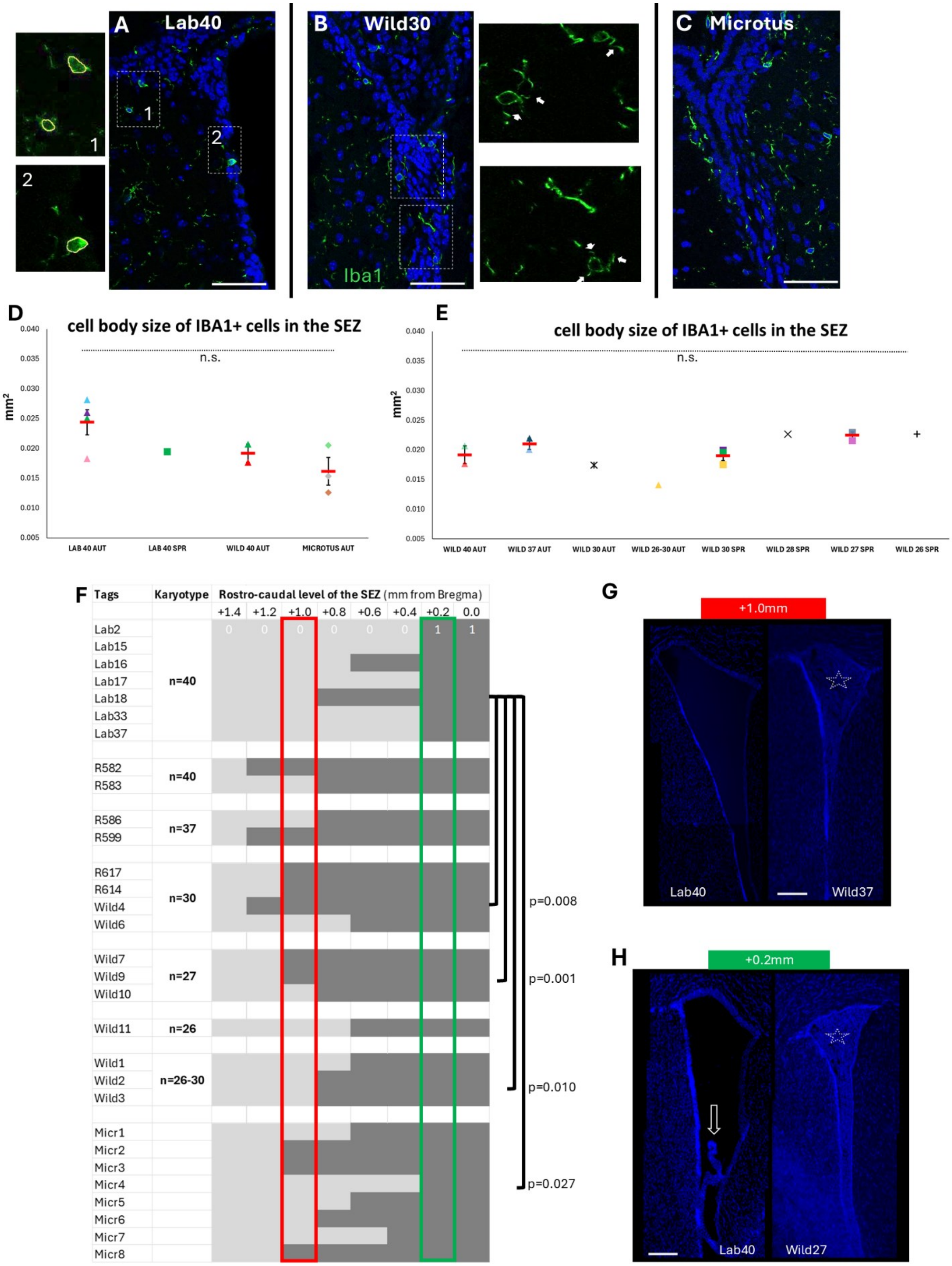
A Lab40
1
2
B Wild30
Iba1
C Microtus
D
cell body size of IBA1+ cells in the SEZ
n.s.
mm²
0.040
0.035
0.030
0.025
0.020
0.015
0.010
0.005
LAB 40 AUT
LAB 40 SPR
WILD 40 AUT
MICROTUS AUT
E
cell body size of IBA1+ cells in the SEZ
n.s.
WILD 40 AUT
WILD 37 AUT
WILD 30 AUT
WILD 26-30 AUT
WILD 30 SPR
WILD 28 SPR
WILD 27 SPR
WILD 26 SPR
F
Tags
Karyotype
Rostro-caudal level of the SEZ (mm from Bregma)
+1.4 +1.2 +1.0 +0.8 +0.6 +0.4 +0.2 0.0
0 0 0 0 0 0 1 1
Lab2
Lab15
Lab16
Lab17
Lab18
Lab33
Lab37
n=40
R582
R583
n=40
R586
R599
n=37
R617
R614
Wild4
Wild6
n=30
Wild7
Wild9
Wild10
n=27
Wild11
n=26
Wild1
Wild2
Wild3
n=26-30
Micr1
Micr2
Micr3
Micr4
Micr5
Micr6
Micr7
Micr8
p=0.008
p=0.001
p=0.010
p=0.027
G
+1.0mm
Lab40
Wild37
H
+0.2mm
Lab40
Wild27

Supplemental Figure 4. Effects of habitat, species, seasons and of chromosome numbers on the population of microglial cells and on the size of the choroid plexus

(A-C) Microphotographs of the SEZ from brain sections immunostained for Iba1 (in green, to mark microglial cells). Note in “A” an indicative photo from a Lab40 mouse brain, showing examples of microglial cells and how their cell body was delineated to measure size (inboxes 1 and 2) using FiJi. In the inboxes of image “B”, from a Wild30 mouse brain, examples of microglial cell processes are shown (indicated by white arrows). In “C” an example of the same area and immunostaining in a *Microtus* brain. (D, E) Scatter plot graphs of the cell body size (in mm^2) of Iba1+ cells within the SEZ of different rodent populations, according to life conditions (lab or wild), seasons, species and numbers of chromosomes. [Individual values are shown with different colours and shapes, as explained in Suppl. Figure 1. Mean values are shown with red lines and error bars depict the SEM. Comparisons were performed using one-way ANOVA followed by Tukey’s *post-hoc* analysis. “n.s.”: not significant]. (F) Raw data of the histological analysis of brain tissue sections, across the rostro-caudal axis based on the mouse brain atlas (Paxinos and Franklin, 2019), recording the presence of the choroid plexus. The plexus’s presence is depicted with grey and the section was given “1” point, while its absence is depicted with white and was attributed “0” points (as exemplified for the first row). The average score for each animal group was calculated and statistical analysis was performed using one-way ANOVA [overall $F=3.70$ and $p=0.009$; for groups with $n \geq 3$, post hoc analysis was performed, and significant results are shown]. (G,H) Low magnification composite microphotographs of DAPI-stained tissue sections showing a rostral (in G) and a caudal (in H) level. Note the presence of choroid plexus filling the ventricle (indicated by a star) in wild animals, and only a small fragment of the choroid plexus at the ventral floor of the ventricle in a Lab mouse in “H” (indicated by the white arrow) [scale bars: 200μm].

Supplemental Table 1. Details of the wild animals used in the study.

The collection sites are shown in Suppl. Figure 1. “*M.m.dom*”: *Mus musculus domesticus*. In gender, M= Male, F= Female. Karyo= karyotype, showing the diploid numbers; Fundamental numbers, for Mus=40, for Microtus=46. Date of sacrifice in the form: DD-MM-YY. “W.B.L.”: whole body length. “T.L.”: tail length. “E.L.”: ear length. “H.F.L.”: hind foot length

Animal number	Location of collection (Greece)	Animal species	Gender	Karyotype (2n)	Date of Sacrifice	Process of sacrifice	Cell Culture	Histology	Weight (g)	Whole Body Length (mm)	Tail Length (mm)	Ear Length (mm)	Hind Foot Length (mm)
R581	Antirrio, Aitoloakarnania	*Mus musculus domesticus*	M	40	6-11-2015	i.v.p. (saline)	-	total brain	14.71	167	87	15	18
R582	Marathias, Fokida	*Mus musculus domesticus*	M	40	12-11-2015	i.v.p. (saline)	-	total brain	12.05	146	75	14	15
R583	Makyneia, Aitoloakarnania	*Mus musculus domesticus*	F	40	12-11-2015	i.v.p. (saline)	-	total brain	9.76	137	73	12.5	17
R584	Trikorfo, Aitoloakarnania	*Mus musculus domesticus*	M	40	12-11-2015	i.v.p. (saline)	-	total brain	13.25	160	85	14	17
R586	Trikorfo, Aitoloakarnania	*Mus musculus domesticus*	F	37	13-11-2015	i.v.p. (saline)	-	total brain	15.4	166	86	14.5	17
R597	Trikorfo, Aitoloakarnania	*Mus musculus domesticus*	F	37	10-12-2015	i.v.p. (saline)	-	total brain	8.98	128	67	14	16
R599	Trikorfo, Aitoloakarnania	*Mus musculus domesticus*	M	37	11-12-2015	i.v.p. (saline)	-	total brain	8.45	127	64	12	16
R600	Trikorfo, Aitoloakarnania	*Mus musculus domesticus*	M	37	11-12-2015	i.v.p. (saline)	-	total brain	8.27	123	66	12.5	17
R613	Elekistra, Achaia	*Mus musculus domesticus*	F	30	26-2-2015	i.v.p. (saline)	-	total brain	23.44	175	92	16	17
R614	Elekistra, Achaia	*Mus musculus domesticus*	F	30	2-3-2016	i.v.p. (saline)	-	total brain	16	155	79	13	15
R617	Elekistra, Achaia	*Mus musculus domesticus*	F	30	3-3-2016	i.v.p. (saline)	-	total brain	10.69	147	82	14.5	16
R619	Elekistra, Achaia	*Mus musculus domesticus*	M	30	8-3-2016	i.v.p. (saline)	-	total brain	9.14	134	71	12	17
R625	Argyra, Achaia	*Microtus thomasi*	F	40	31-3-2016	i.v.p. (saline)	-	total brain	9.04	86	17	8	14
R626	Argyra, Achaia	*Microtus thomasi*	M	40	31-3-2016	i.v.p. (saline)	-	total brain	9.26	89	17	8	14
R627	Argyra, Achaia	*Microtus thomasi*	M	40	31-3-2016	i.v.p. (saline)	-	total brain	25.38	126	25	10	15
Wild Mus 1	Mavroneri, Voiotia	*Mus musculus domesticus*	M	26-30	21-10-2021	i.v.p. (saline)	left hemisphere	right hemisphere	13.59	168	88	14	16
Wild Mus 2	Mavroneri, Voiotia	*Mus musculus domesticus*	F	26-30	21-10-2021	i.v.p. (saline)	right hemisphere	left hemisphere	13.89	149	75	11	17
Wild Mus 3	Mavroneri, Voiotia	*Mus musculus domesticus*	F	26-30	21-10-2021	i.v.p. (saline)	-	right hemisphere	15.97	171	85	11	16
Wild Mus 4	Mavroneri, Voiotia	*Mus musculus domesticus*	M	30	21-10-2021	i.v.p. (saline)	right hemisphere	left hemisphere	17.26	169	85	14	16
Wild Mus 5	Mavroneri, Voiotia	*Mus musculus domesticus*	M	26-30	21-10-2021	i.v.p. (saline)	left hemisphere	right hemisphere	13.4	152	74	11	15
Wild Mus 6	Mavroneri, Voiotia	*Mus musculus domesticus*	M	30	10-3-2022	i.v.p. (saline)	-	left hemisphere	17.88	177	98	14.5	17
Wild Mus 7	Mavroneri, Voiotia	*Mus musculus domesticus*	F	27	10-3-2022	i.v.p. (saline)	-	right hemisphere	13.13	150	80	14	16
Wild Mus 8	Mavroneri, Voiotia	*Mus musculus domesticus*	M	28	10-3-2022	i.v.p. (saline)	right hemisphere	left hemisphere	20.42	175	92	14.5	17
Wild Mus 9	Mavroneri, Voiotia	*Mus musculus domesticus*	M	27	11-3-2022	i.v.p. (saline)	left hemisphere	right hemisphere	13.9	---	85	14	17
Wild Mus 10	Mavroneri, Voiotia	*Mus musculus domesticus*	F	27	11-3-2022	i.v.p. (saline)	right hemisphere	left hemisphere	18	170	89	14.5	17
Wild Mus 11	Mavroneri, Voiotia	*Mus musculus domesticus*	M	26	11-3-2022	i.v.p. (saline)	left hemisphere	right hemisphere	17.24	174	92	14.5	17
Wild Mus 13	Mavroneri, Voiotia	*Mus musculus domesticus*	M	26-30	11-10-2023	i.v.p. (saline)	both hemispheres	no	18.25	166	83		
Microtus 1	Kydonies, Achaia	*Microtus thomasi*	M	40	24-11-2021	i.v.p. (saline)	right hemisphere	left hemisphere	25.78	119	25	6	15
Microtus 2	Kydonies, Achaia	*Microtus thomasi*	F	40	24-11-2021	i.v.p. (saline)	left hemisphere	right hemisphere	23.71	120	18	7	15
Microtus 3	Kydonies, Achaia	*Microtus thomasi*	M	40	24-11-2021	i.v.p. (saline)	right hemisphere	left hemisphere	25.3	119	16	8	15
Microtus 4	Kydonies, Achaia	*Microtus thomasi*	M	40	24-11-2021	i.v.p. (saline)	left hemisphere	right hemisphere	22.91	119	16	8	15
Microtus 5	Kydonies, Achaia	*Microtus thomasi*	F	40	24-11-2021	i.v.p. (saline)	right hemisphere	left hemisphere	23.67	118	19	7	16
Microtus 6	Kydonies, Achaia	*Microtus thomasi*	M	40	30-11-2021	i.v.p. (saline)	-	right hemisphere	24.14	118	19	9	15
Microtus 7	Kydonies, Achaia	*Microtus thomasi*	M	40	30-11-2021	i.v.p. (saline)	right hemisphere	left hemisphere	23.5	120	16	9	15
Microtus 8	Kydonies, Achaia	*Microtus thomasi*	M	40	30-11-2021	i.v.p. (saline)	-	left hemisphere	24.93	115	19	8	15
Microtus 9	Kydonies, Achaia	*Microtus thomasi*	M	40	30-11-2021	i.v.p. (saline)	left hemisphere	right hemisphere	20.34	117	21	9	18

Supplemental Table 2. Details of the statistical comparisons of in vivo data

Summary of the comparisons that were performed using the data generated by the immunohistochemical analysis of tissue sections. The table is separated according to the animal groups compared (based on habitat, species, season and number of chromosomes) and the brain area analysed (SEZ or CC). Two main types of statistical analyses were performed: one-way ANOVA or Pearson's correlation. Variables and the respective statistical values shown I grey returned non-significant results. In the last column we refer the Figure in which the data are shown in the form of graph.

	Variables	**Statistical tools & metrics**	***Location in the manuscript***
The effects of **habitat, seasons** and **species**<br>**Wild40** (control), **Lab40**^{AUTUMN}, **Lab40**^{SPRING}, **Microtus**^{AUTUMN}, **Microtus**^{SPRING}			
		One-way ANOVA (Tukey's *post-hoc* analysis shown on graphs)	
**In the SEZ**	*PCNA density*	F=4.631, p<0.001	*Fig 1F*
	Dcx density	F=3,954, p=0.001	*Fig 1G*
	%PCNA	F=4.060, p=0.001	*Sup Fig 2D*
	%Dcx	F=5.830, p<0.001	*Sup Fig 2E*
	Olig2 density	F=1.727, p=0.214	*Fig 3B*
	Iba1 density	F=0.774, p=0.645	*Fig 3C*
	Iba1 cell body size	F=2.386, p=0.089	*Sup Fig 4D*
	Supporting cell density	F=1.759, p=0.105	*Sup Fig 3A*
**In the CC**	*Olig2 PCNA density*	F=1.609, p=0.261	*Fig 4C*
	Olig2 PCNA/Olig2	F=1.442, p=0.364	*Fig 4E*
	Sox2 density	F=4.382, p=0.033	*Fig 4G*
	Iba1 density	F=2.657, p=0.081	*Fig 4J*
The effects of **chromosome numbers** in AUTUMN<br>**Wild40** (control), **Wild37**, **Wild30**, **Wild26-30**<br>**correlation with "2n"** *and* comparison vs **Lab40**			
		Pearson's correlation	
**In the SEZ AUTUMN**	*PCNA density*	ρ=0.965	*Fig 2C*
	Dcx density	ρ=0.961	*Fig 2E*
	%PCNA	ρ=0.934	*Sup Fig 2F*
	%Dcx	ρ=0.936	*Sup Fig 2H*
	Olig2 density	ρ=0.414	*Fig 3C*

	Iba1 density	ρ=0.395	*Fig 3D*
	Supporting cell density	Not enough power	*Sup Fig 3B*
**In the CC AUTUMN**	*Olig2 PCNA density*	ρ=0.544	*Fig 4D*
	Olig2 PCNA/Olig2	ρ=-0.246	*Fig 4F*
	Sox2 density	ρ=0.706	*Fig 4H*
	Iba1 density	ρ=-0.463	*Fig 4K*
		One-way ANOVA (Tukey's post-hoc analysis vs **Lab40** on graphs)	
**In the SEZ AUTUMN**	*PCNA density*	F=4.631, p<0.001	*Fig 2C*
	Dcx density	F=3,954, p=0.001	*Fig 2E*
	%PCNA	F=4.060, p=0.001	*Sup Fig 2F*
	%Dcx	F=5.830, p<0001	*Sup Fig 2H*
	Olig2 density	F=1.727, p=0.214	*Fig 3C*
	Iba1 density	F=0.774, p=0.645	*Fig 3D*
	Iba1 cell body size	F=2.386, p=0.089	*Sup Fig 4E*
	Supporting cell density	F=1.759, p=-0.105	*Sup Fig 3B*
**In the CC AUTUMN**	*Olig2 PCNA density*	F=1.609, p=0.261	*Fig 4D*
	Olig2 PCNA/Olig2	F=1.442, p=0.364	*Fig 4F*
	Sox2 density	F=4.382, p=0.033	*Fig 4G*
	Iba1 density	F=2.657, p=0.081	*Fig 4K*
The effects of **chromosome numbers** in SPRING<br>**Wild30**, **Wild28**, **Wild27**, **Wild26**<br>**correlation with "2n"** *and* comparison vs **Lab40**			
		Pearson's correlation	
	PCNA density	ρ=0.934	*Fig 2D*
	Dcx density	ρ=0.878	*Fig 2E*

**In the SEZ SPRING**	*%PCNA*	ρ=0.933	*Sup Fig 2G*
	%Dcx	ρ=0.860	*Sup Fig 2I*
	Olig2 density	ρ=-0.032	*Fig 3C*
	Iba1 density	ρ=-0.701	*Fig 3D*
	Supporting cell density	ρ=-0.248	*Sup Fig 3C*
**In the CC SPRING**	*Olig2 PCNA density*	ρ=0.072	*Fig 4D*
	Olig2 PCNA/Olig2	ρ=-0.021	*Fig 4F*
	Sox2 density	ρ=0.995	*Fig 4I*
	Iba1 density	ρ=-0.757	*Fig 4K*
		One-way ANOVA (Tukey's post-hoc analysis vs **Lab40** on graphs)	
**In the SEZ SPRING**	*PCNA density*	F=4.631, p<0.001	*Fig 2D*
	Dcx density	F=3,954, p=0.001	*Fig 2E*
	%PCNA	F=4.060, p=0.001	*Sup Fig 2G*
	%Dcx	F=5.830, p<0001	*Sup Fig 2I*
	Olig2 density	F=1.727, p=0.214	*Fig 3C*
	Iba1 density	F=0.774, p=0.645	*Fig 3D*
	Iba1 cell body size	F=2.386, p=0.089	*Sup Fig 4E*
	Supporting cell density	F=1.759, p=-0.105	*Sup Fig 3C*
**In the CC SPRING**	*Olig2 PCNA density*	F=1.609, p=0.261	*Fig 4D*
	Olig2 PCNA/Olig2	F=1.442, p=0.364	*Fig 4F*
	Sox2 density	F=4.382, p=0.033	*Fig 4I*
	Iba1 density	F=2.657, p=0.081	*Fig 4K*
